# Supplementary material for: Effectiveness of a nurse-led hospital-to-home transitional care intervention for older adults with multimorbidity and depressive symptoms: A pragmatic randomized controlled trial
Source: PLoS One. 2021 Jul 26;16(7):e0254573. doi: 10.1371/journal.pone.0254573 (PMC8312945; doi:10.1371/journal.pone.0254573)
Supplement: S2 Table — (DOCX) [file pone.0254573.s002.docx]

**S2 Table. Group Differences in Patient Experience Outcomes at Baseline and Six-Months (CCQ, IC-PREMs) (n=99)**

| **Questions** | **Baseline**  **Chi-Square**  **(p-value)** | **T2**  **Chi-Square**  **(p-value)** |
| --- | --- | --- |
| 1. To what extent do you agree or disagree with the following statement: "I am given enough opportunity to help **decide on care I receive**" | 0.18 (0.67) | 0.03 (0.85) |
| 2. Do you know **who to contact** if you need to ask questions about your condition(s) or treatment(s)? | 1.31 (0.25) | 0.40 (0.53) |
| 3. If you have questions, **when can you contact** the people treating and caring for you? | 0.41 (0.52) | 1.48 (0.22) |
| 4. Do you feel the people treating or caring for you **understand about you** and your conditions? | 1.03 (0.32) | 0.18 (0.67) |
| 5. To what extent do you agree or disagree with the following statement... "In the last 6 months, health, and social care staff have **given me information** about other services that are available to someone in my circumstances, including support organizations." | 1.82 (0.18) | 4.88 (0.03) |
| 6. Were you **involved** as much as you wanted to be in decisions about your care and support? | 0.00 (0.98) | 0.12 (0.72) |
| 7. To what extent do you agree or disagree with the following statement... "I can **see the impact** of my involvement on how my care is delivered”? | 1.51 (0.22) | 0.18 (0.68) |
| 8. Do health and social care services help you to **live the life you want** as far as possible? | 0.20 (0.65) | 1.73 (0.19) |
| 9. When health or social care staff **plan** care or treatment for you, does it **happen?** | 3.0 (0.08) | 0.14 (0.71) |
| 10. To what extent do you agree or disagree with the following statement... "My care and support is **reviewed** as often as it should be." | 0.15 (0.70) | 0.31 (0.58) |
| 11. To what extent do you agree or disagree with the following statement... "My **medicines are thoroughly reviewed** as often as they should be." | 0.00 (0.94) | 3.36 (0.07) |
| 12. Do you have a named health or social **care professional who coordinates** your care and support? | 1.9 (0.17) | 0.44 (0.51) |
| 13. Do all the different people treating and caring for you **work well together** to give you the best possible care and support? | 1.17 (0.28) | 1.80 (0.18) |
| 14. Have all your **needs** been assessed? | 0.00 (0.10) | 0.50 (0.48) |
| 15. Were your **family or caregiver involved** in decisions about your care and support as much as you wanted them to be? | 0.13 (0.72) | 0.01(0.90) |
| 16, **Overall,** do you feel that your caregiver/family has had as much **support** from health and social services as they needed? | 0.06 (0.80) | 0.02 (0.87) |
