# Supplementary material for: Effectiveness of a nurse-led hospital-to-home transitional care intervention for older adults with multimorbidity and depressive symptoms: A pragmatic randomized controlled trial
Source: PLoS One. 2021 Jul 26;16(7):e0254573. doi: 10.1371/journal.pone.0254573 (PMC8312945; doi:10.1371/journal.pone.0254573)
Supplement: S1 File — (DOCX) [file pone.0254573.s003.docx]

**S1 File. Study protocol approved by the McMaster University Hamilton Integrated Research Ethics Board**

**A pragmatic effectiveness-implementation trial to evaluate a hospital-to-home transitional care intervention compared to usual care for older adults with multiple chronic conditions and depression**

**Principal Investigator:** Maureen Markle-Reid, PhD (McMaster)

**Co-Principal Investigator:** Carrie McAiney, PhD (Waterloo)

**Co-Investigators:** David Price, MD (McMaster, Hamilton Health Sciences)

Ruta Valaitis, PhD (McMaster)

Jenny Ploeg, PhD (McMaster)

Rebecca Ganann, PhD (McMaster)

Kathryn Fisher, PhD (McMaster)

Nick Kates, MD (McMaster)

John Lavis, MD, PhD (McMaster)

Lehana Thabane, PhD (McMaster)

Amiram Gafni, PhD (McMaster)

Isabelle Vedel, MD-MPH, PhD (McGill)

Wayne Warry, PhD (Laurentian)

Alain Gauthier, PhD (Laurentian)

Diana Urajnik, PhD (Laurentian)

Patricia Wilson, PhD (Kent)

Kirsten Krull (Hamilton Health Sciences)

Janet McElhaney, MD (Health Sciences North)

Cheryl Williams, PhD (Joseph Brant Hospital)

Bill Johnson (Patient/Caregiver Stakeholder)

Gail Heald-Taylor (Patient/Caregiver Stakeholder)

Lawrence Ogden (Patient/Caregiver Stakeholder)

Patricia Reid (Patient/Caregiver Stakeholder)

Research Coordinator: [to be hired]

Trainees: Carly Whitmore, BScN, MSc (McMaster)

Lead Site: McMaster University

Partner Sites: Hamilton Health Sciences

Joseph Brant Hospital

Health Sciences North

Laurentian University

University of Waterloo

**Sponsor:** Ontario SPOR Support Unit (OSSU)

Labarge Foundation

**ClinicalTrials.gov ID:** NCT03157999

**TABLE OF CONTENTS**

| SUMMARY .................................................................................................................... | 4 |
| --- | --- |
| BACKGROUND AND RATIONALE ................................................................................... | 4 |
| Depression in Older Adults with Multiple Chronic Conditions ......................... | 4 |
| Transitional Care .............................................................................................. | 4 |
| Governance Structures ..................................................................................... | 5 |
| RESEARCH QUESTIONS ................................................................................................. | 6 |
| METHODS ..................................................................................................................... | 6 |
| Study Design ..................................................................................................... | 6 |
| Research Sites .................................................................................................. | 6 |
| Participants ....................................................................................................... | 6 |
| Recruitment and Informed Consent ................................................................. | 7 |
| Randomization ................................................................................................. | 10 |
| Intervention ……................................................................................................ | 10 |
| Capacity-Building and Training....…………………………………………………………………. | 11 |
| Measures and Data Collection ......................................................................... | 12 |
| DATA ANALYSIS ............................................................................................................ | 14 |
| REFERENCES ................................................................................................................. | 16 |
| LIST OF ABBREVIATIONS ............................................................................................... | 18 |
| REVISION HISTORY ....................................................................................................... | 19 |
| APPENDICES * |  |
| *Appendix A: Governance Structures* |  |
| *Appendix B: Qualifying Comorbid Conditions* |  |
| *Appendix C: Study Timeline* |  |
| *Appendix D: Participant Flow Diagram* |  |
| *Appendix E: Screening Script* |  |
| *Appendix F: Eligibility Questionnaire* |  |
| *Appendix G: Participant Contact Information Form* |  |
| *Appendix H: Participant Consent Form* |  |
| *Appendix I: Participant Interview (T1)* |  |
| *Appendix J: Research Assistant Telephone Scripts* |  |
| *Appendix K: ICES Research Plan* |  |
| *Appendix L: Nurse Telephone Script* |  |
| *Appendix M: Variables and Measures* |  |
| *Appendix N: Participant Interview (T2, T3)* |  |
| *Appendix O: Caregiver Self-Administered Questionnaire* |  |
| *Appendix P: Care Transition Coordinator Interview 1 Guide* |  |
| *Appendix Q: Care Transition Coordinator Interview 2 Guide* |  |
| *Appendix R: Key Informant Consent Form* |  |
| *Appendix S: Participant and Caregiver Post-Intervention Interview*  *Appendix T: Hospital Recruiter Role*  *Appendix U: Graphical Depiction of Trial Components*  *Appendix V: Study Information Sheet*  *Appendix W: Research Partner Questionnaire*  *Appendix X: CAB Interview 1 Guide*  *Appendix Y: Appointment Reminder Card*  *Appendix Z: Steering Committee Interview 1 Guide*  *Appendix AA: Physician Letter*  *Appendix AB: Recruitment Poster for all Allied Health Professionals*  *Appendix AC: Participant Consent to Share Information – CTC & Circle of Care*  *Appendix AD: Participant Consent to Share Information – CTC Referrals*  *Appendix AE: CAST Discharge Summary*  *Appendix AF: Participant & Caregiver Consent Form – Post-Intervention Interview*  *Appendix AG: Research Partner Questionnaire Email Invitation*  *Appendix AH: Caregiver Letter of Information*  *Appendix AI: CAB Interview 2 Guide*  *Appendix AJ: Patient/Caregiver Research Partners – Interview 1 Guide* |  |

*All appendices are attached separately

**SUMMARY**

Few older adults diagnosed with depression receive adequate treatment, despite the fact that it is one of the most common mental health conditions affecting this population (1). Transitioning from hospital to home is a risk factor for depression, particularly in older adults who have multiple health problems and few social supports. Primary care is a critical safety net, yet low rates of identification and treatment of depression exist in this setting. Additionally, less than one-third of those hospitalized have follow-up visits with their doctor within one week of leaving the hospital (3). The aim of this study is to determine the effects and costs of a 6-month hospital-to-home support program for older adults with multiple chronic conditions (MCC) and symptoms of depression.

A pragmatic randomized controlled trial design will be used to compare patients receiving the intervention plus usual care to a usual care-only control group. The intervention will be based on one that was successfully tested in a pilot study, and may include additional components that have received support in the transitional care literature. Components of the program include home visits, telephone follow-up, and nurse-led care coordination. The overall goal is to improve health outcomes in older adults with depressive symptoms and other health problems. Patients, caregivers, providers and researchers will work side-by-side to implement and test the program in diverse settings to inform rapid scale-up across Ontario.

**BACKGROUND AND RATIONALE**

**Depression in Older Adults with Multiple Chronic Conditions**

One in three Canadian seniors have MCC, and account for 40% of health care use. Older adults with MCC report poorer quality of life, higher health service use/costs, and higher risk of adverse events (e.g., hospitalization), compared to those with single conditions. Furthermore, these individuals are frequently in the position of having to navigate a patchwork of costly services across multiple providers and settings (2).

Depression is one of the most common mental health problems affecting older adults using home care services, yet only 12% of those with depression receive adequate treatment (1). Furthermore, an estimated one-third of Ontarians hospitalized for depression do not receive follow-up care within 30 days post-discharge (3). Older adults are more vulnerable to depression due to the increased likelihood of MCC, a known risk factor, as well as limitations on physical functionality and social interaction (4). Depressive symptoms are associated with a wide range of chronic conditions, such as heart disease and diabetes (5), and present challenges for self-management as well as the treatment and management of other conditions (6). Recent evidence suggests that the coexistence of mental and physical problems significantly increases unplanned hospital visits and use of other health services (7), yet most intervention studies of older adults with MCC do not address mental health.

**Transitional Care**

Transitioning from hospital to home is a known risk factor for depression, particularly in older adults who have MCC and few social supports. Deficits in quality of care to support transitions from hospital to community result in about 20% readmission rates (8) and preventable adverse events (9). Service gaps are evident, with only 29% of Ontarians seeing a primary care physician within seven days post-discharge (10). Quality of care and patient safety are compromised because of medication errors, incomplete information transfer, and lack of follow-up care. Older adults are often unprepared to self-manage, forced to navigate a constantly shifting landscape of providers and sites, receive conflicting advice regarding co-morbidity management, experience difficulties accessing services, and have limited input into their care (11).

There is limited evidence on how to provide transitional care to older adults with MCC and depressive symptoms. Most trials evaluating transitional care interventions have (a) used weak designs, (b) excluded older adults with MCC, (c) focused on single conditions and omitted mental health, and/or (d) provided limited information on cost and implementation strategies (12-15). Furthermore, most studies have focused on the effect of transitional care interventions on re-hospitalization rates with less attention paid to self-management, other patient-relevant outcomes, or primary care (13, 16-18).

Implementing transitional care interventions for older adults with depressive symptoms and MCC is a pressing concern since older adults with depression face persistent health disparities. The **Community Assets Supporting Transitions (CAST)** research team seeks to address these health inequities, and improve health outcomes in this vulnerable and under-served population by developing and implementing an intervention that will improve patients’ self-management ability, support their families and caregivers, and build capacity for primary care and other health and non-health providers to collaborate in delivering home and community services.

The proposed study builds on our pilot study that evaluated a nurse-led intervention for older adult home care clients with MCC and depressive symptoms and demonstrated that the intervention was feasible and effective in reducing depressive symptoms (19). In the pilot, 60% of the 142 participants had at least one hospital admission in the 6 months prior to baseline, suggesting there is a need for an intervention to support older adults making this transition (19, 20).

**Governance Structures**

A set of structures will be established to guide and support this research (see Appendix A):

1. *Community Advisory Boards* (CABs) in each of the three communities to oversee and tailor the implementation of the intervention in each community.
2. *Patient and Caregiver Ad Hoc Group* which will be brought together for specific tasks throughout the study, including identifying study outcome measures that are meaningful to patients and caregivers, reviewing data collection tools, and advising on the sharing of study findings.

Patients and caregivers are represented within each of these structures, and will receive training relevant to the research study as well as ongoing support to ensure they are able to meaningfully participate. Patients and caregivers will also receive a stipend for $75 for each meeting they participate in. (This is intended to compensate time preparing for, traveling to, and attending meetings.)

The CABs will also include representatives from health care organizations (e.g., hospital, CCAC), community support services, and non-health organizations (e.g., municipal government).

**RESEARCH QUESTIONS**

The overall aim of the study is to improve care transitions in older adults with MCC and depressive symptoms. The project will address three research questions:

1. What is the effect of a new, nurse-led hospital-to-home transitional care intervention compared to usual care on health outcomes and costs for older adults with MCC and depressive symptoms?
2. How is a care transition intervention adapted and implemented in diverse settings?
3. What is required to sustain and scale up the intervention?

We hypothesize that the intervention will result in improvements in health outcomes and reduced use of expensive health services compared to usual care at no additional cost, from a societal perspective.

**METHODS**

**Study Design**

The proposed study design is a pragmatic randomized controlled trial (RCT). A pragmatic design applies RCT methodology in actual care settings to better inform decisions on the likely benefits, harms, and costs of real world implementation (21). The design is further classified as a Type II hybrid effectiveness-implementation study (22), which assigns equal weight to assessing program effectiveness and implementation.

**Research Sites**

The intervention will be tested in three regions that reflect diversity across Ontario: 1) Sudbury, 2) Burlington, and 3) Hamilton.

**Participants**

***A) Effectiveness***: Participants for the trial will be recruited from hospitals within each of the three research sites. Each site will recruit 72 patients, for a total sample size of 216. Consistent with our previous study (19), we anticipate that approximately 50% of individuals screened will be eligible to enter the study and that 40% of those will agree to be in the study. Thus, to enroll 216 participants, approximately 1,080 patients will be screened, 540 of whom will be deemed eligible to participate. Sample size justification and subgroup analyses are described below (see DATA ANALYSIS).

*Inclusion Criteria:*

A patient will be considered eligible for the trial if he/she

- Is an adult, age 65 or older;
- Is planned for discharge from hospital to the community (this includes retirement homes and transitional care beds);
- Self-reports having a diagnosis of at least two chronic conditions (see list of chronic conditions in Appendix B);
- Is experiencing depressive symptoms, assessed using the 2-item version of the Patient Health Questionnaire (PHQ-2);
- Lives within one of the study regions (Sudbury, Burlington, or Hamilton), and is not planning to move out of the region during the trial (defined as a one-year period);
- Is capable of providing informed consent, or has a substitute decision-maker who is able to provide informed consent on his/her behalf; and
- Is competent in English, or has an interpreter who is competent in English. At the Sudbury site, French-speaking individuals (or those with French-speaking interpreters) will also be eligible.

*Exclusion Criteria:*

A patient will be excluded from the trial if he/she

- Is being discharged from the hospital to a long-term care home or tertiary care.

The adult caregiver(s) of trial participants, defined as family members or friends who assist with the management of their day-to-day activities by providing emotional, financial or other support, will also be invited to participate in the trial.

***B) Implementation of the Intervention***:

A variety of individuals will be invited to participate in evaluating over time the implementation component of the study, which includes the patient/caregiver engagement strategy. This includes: i) members of the CABs; ii) the Care Transition Coordinators or CTCs (i.e., the intervention nurses); iii) key informants who are working collaboratively with the CTCs to implement the intervention (the specific organizations represented are likely to vary from site to site, but are likely to include representatives from the participating hospitals and the CCAC); and iv) a sample of patients and caregivers that receive the intervention.

**Recruitment and Informed Consent**

***A) Effectiveness***: Recruitment will take place in participating hospitals at each of the study sites. These hospitals will be Health Sciences North (HSN) in Sudbury, Joseph Brant Hospital (JBH) in Burlington, and Hamilton General Hospital (HGH) in Hamilton.

Please refer to Appendix C for a study timeline and Appendix D for a participant flow diagram. Screening for study eligibility will be done prior to discharge by hospital-based recruiters (see Appendix T, Hospital Recruiter Role). The recruiter will regularly liaise with hospital staff to identify patients who are interested in being approached about the study. Hospital staff will be familiar with the eligibility criteria (e.g., 65 years of age or older, not planned for discharge to long-term care), and will initially approach patients who are potentially eligible. Once a member of a patient’s circle of care has obtained permission for the recruiter to approach the patient, the recruiter will introduce the study to the patient, following the Screening Script (see Appendix E). If patients agree to be screened, the recruiter will administer the Eligibility Questionnaire (see Appendix F). The Recruiter will confirm whether or not each patient is eligible by completing and signing off on the Eligibility Questionnaire. Eligible patients who agree to be contacted by a Research Assistant upon discharge from hospital will receive a Study Information Sheet (see Appendix V) to take home with them.

The Recruiter will fax completed Eligibility Questionnaires and Participant Contact Information Forms (see Appendix G) to the Research Coordinator (RC) at McMaster within 24 hours of screening. Hard copies of these forms will be stored in a secure location (a locked cabinet in a locked office) at each hospital. At the conclusion of recruitment at each site, the hard copy materials will be couriered to the RC at McMaster.

The recruiter will maintain a password-protected electronic Recruitment Log that tracks all patients who are approached about the study. This log will be stored on a secure hospital computer, and it will include the following information: patient’s name, date of birth, date on which they were approached by the Recruiter and study eligibility information This information will be stored in the log only for the duration of the recruitment period so that the recruiter can cross-reference the file to determine which patients have previously been approached for the study and to avoid collecting information from and re-approaching the same patients multiple times, with the goal of reducing burden on the patient population. The recruiter will only be approaching (and, therefore, recording information about) patients who have agreed to be approached about the study. The recruiter will not share the Recruitment Log with anyone, and the log will be deleted once recruitment has been completed.

The recruiter will not be accessing any patient information prior to approaching patients and obtaining their permission to access their records (in a limited way). Information collected from the patient record (with patient permission) will include date of birth, reason for admission to hospital, primary diagnosis, and (if available) where the patient is to be discharged to (e.g., home, long-term care, transitional bed, etc.). This information, along with patient self-reported information collected on the Eligibility Questionnaire (Appendix F) will be faxed to the main study site, regardless of whether or not the patient is eligible. However, the data will be de-identified and the names of ineligible patients will never be shared with the main study site. These data will enable the researchers to describe the sample, assess the representativeness of the sample, and inform intervention scale-up.

The Recruiter will additionally maintain a separate electronic Discharge Log that tracks discharge status only for those patients who are deemed eligible and who agree to be contacted by the RA. This de-identified log will only contain each eligible patient’s recruitment number, anticipated date of discharge, and confirmed date of discharge. It will be shared with the RC at the McMaster site via email in order to ensure that the coordinating site has up-to-date information about patient discharges, thus enabling timely follow-up with patients post-discharge. The Recruiter will regularly liaise with a unit clerk, or designated unit representative, to confirm and update the discharge status of each patient that he/she is tracking.

Teleconference calls will be held every other week for the duration of the recruitment period between each of the Recruiters and the RC at McMaster. These calls will be an opportunity to discuss recruitment issues, provide updates, and engage in oversight and ongoing training.

The RC will regularly provide confirmed patient discharge dates to an RA working within the community. As soon as possible following the patient’s discharge, the RA will call the patient to review the consent form (Appendix H) with them. The patient will have the option of scheduling a phone call or in-home visit to complete the initial consent process. If the patient chooses not to participate, the RA will thank them for their time and record the reason for refusal, if one is provided. Three attempts will be made to contact the patient at different times of day. If the patient cannot be reached, 3 attempts will be made to get in touch with an alternate contact (if the patient provided one at the time of recruitment). If the RA is not able to contact the patient or alternate contact, no further attempts will be made and the participant will be recorded as “declined” (unable to reach).

During the first scheduled visit/call, prior to reviewing the consent form with the patient, the RA will establish the patient’s cognitive capacity to provide informed consent on his/her own behalf, by administering the Short Portable Mental Status Questionnaire (SPMSQ). If the patient achieves a score of 5 or more on the SPMSQ, then the RA will obtain the patient’s written informed consent to participate in the trial. Once consent is provided, the participant will be enrolled in the trial and he or she will be asked to complete the Baseline (T1) Assessment.

If the patient scores less than 5 on the SPMSQ, the patient’s SDM will be approached to provide consent and complete the questionnaires on their behalf. The SDM may be a caregiver, as defined earlier, (i.e., family member or friend) of the patient. The SDM is not required to be a formally-appointed decision maker (i.e., is not required to hold power of attorney for personal care).

If an SDM is present at the time of the interview, and is willing to provide consent and complete the questionnaires on behalf of the patient, the RA will establish the SDM’s cognitive capacity to provide informed consent by administering the SPMSQ. If the SDM achieves a score of 5 or more, then the RA will obtain the SDM’s written informed consent for the patient to participate in the study and complete the Baseline (T1) Assessment. If the SDM scores less than 5 on the SPMSQ, then the interview will not proceed.

If an SDM is not present at the time of the interview, then the visit/call will be rescheduled to include an SDM. If the patient is unable to identify someone to act as his/her SDM, then the patient will not be eligible to participate.

If the consent process is completed over the phone, it will be audio-recorded to facilitate data verification. The RA will sign the consent form to indicate that he/she fully reviewed the form with the patient, and the RA will send a copy of the consent form to the participant for him/her to keep. The consent statement portion of the consent form, signed by the RA, will be sent to the RC following the call. If the consent process is completed in person, the participant, or a substitute decision-maker, will sign the consent form, and will be provided a copy to keep.

The consent form will also include a separate section seeking the participant’s permission to link his/her data to the Institute for Clinical Evaluative Sciences (ICES) database. (See Appendix K for ICES Research Plan). Participants who opt out of ICES data linking will still be able to participate in the trial.

The RA will administer the SPMSQ prior to each follow-up interview at 6-months (T2) and 12-months (T3) with the patient, to establish his or her mental capacity to complete the assessments. In the event that the patient fails to provide a sufficient number of correct responses on the SPMSQ, then an SDM will be invited to complete the assessment on the patient’s behalf; following the SDM’s successful completion of the SPMSQ, and signed informed consent, as described above.

The Baseline Assessment data, as well as the data from the 6- and 12-month follow-up assessments, will be inputted by the RA into Lime Survey. Lime Survey is a data collection software that stores data on secure McMaster University servers.

***B) Implementation of the Intervention***:

Organizations that are working collaboratively with the Care Transition Coordinators (CTCs) to implement the intervention (e.g., hospitals, CCAC) will be identified in each site. Staff members from each of these organizations that are working with the CTCs will be invited to participate in a focus group.

The members of the CABs will also be invited to participate in focus groups because of their role in overseeing and adapting the implementation of the intervention in their communities, as well as the implementation of the study governance structures (See Appendix X: CAB Interview 1 Guide). Research team members that are part of the CABs will not participate in order to reduce the potential of biased responses. Patient and caregiver representatives on the CABs will be invited to participate in separate focus groups to ensure their unique perspectives are captured and to address any potential power imbalances that may limit their ability to share opinions.

In terms of recruiting a sample of patients and caregivers who received the intervention to complete a post-intervention interview, approximately 5 patients and 2 caregivers will be selected per site, for a total of approximately 21 interviews. Patients and caregivers will be selected purposefully by the RC and invited to participate. Selection will be based on complexity of care (medical and social needs) and dose of intervention (minimum dose received vs. maximum).

**Randomization**

***A) Effectiveness***: After informed consent is obtained, the participant will be randomized either to the 6-month intervention (CAST) group or the usual care control group. Computerized software (RedCap), administered by an independent organization, will be used to randomize participants into the intervention or usual care groups within each region using a 1:1 ratio. The RAs, who will conduct subsequent assessments with the participants, will remain blind to the participants’ allocation for the duration of data collection.

**Intervention**

Participants in the intervention group will receive the CAST hospital-to-home transition intervention in addition to usual care (see Appendix U for a graphical depiction of trial components). Intervention duration is expected to be 6 months. A full-time registered nurse (RN) will be hired within each region to function as a CTC who works collaboratively with one local hospital and other health and non-health representatives to deliver the intervention. See below for a description of the CTC’s activities.

Once a participant has been randomized to the intervention group, the RC will provide that participant’s information to the CTC to enable them to contact the participant and begin delivery of the intervention. (See Appendix L for Nurse Telephone Script). The participant’s name and contact information will be shared with the CTC by the RC via MacDrop, a data sharing software that houses data on secure McMaster University servers.

The CTC will contact intervention participants by telephone within 1 month post-discharge. If needed, the CTC will make up to 3 attempts to contact the participant in order to schedule the first home visit. If the nurse is unable to reach the participant, he/she will attempt to reach an alternate contact person (if one was provided by the patient upon recruitment).

Nurse CTCs will deliver the intervention through two modes: home visits (between 1 and 6) and phone calls (minimum of 4). The mode and number of contacts will be tailored to the needs and preferences of each participant. The intervention is likely to include the following general activities, which will also be tailored to the needs and preferences of each participant:

- care coordination and system navigation (including facilitating timely primary care follow-up);
- medication management;
- assessing the needs and risk of the participants (including in-depth assessment of depressive symptoms);
- evidence-based management of depressive symptoms and other chronic conditions to prevent the onset and worsening of other chronic conditions;
- patient and caregiver education; and
- goal setting and problem-solving therapy.

The implementation of the intervention will be reviewed and adapted by the local CABs to address any issues that arise as the intervention is implemented.

**Capacity-Building and Training**

***Recruiters and Research Assistants***

Recruiters at each site will each attend a one-time 4-hour training session, which will be provided by the research team. Oversight (and ongoing training, as needed) will take place as part of regular teleconferences with the RC.

***Care Transition Coordinators***

The CTC at each site will receive training from the research team in intervention delivery and documentation related to intervention implementation. They will also receive training in identifying and managing depressive symptoms. The researchers will share tools for depressive symptom screening, evidence-based behavioural therapies and health promotion strategies effective in addressing depressive symptoms (e.g., social activation theory, problem-solving therapy), and best practices for integrating risk factors in transitional care planning. Oversight (and ongoing training, as needed) will take place as part of regular teleconferences with the RC and research team).

***Governance Structures***

Our research team will provide training, as needed, during CAB meetings to all network members (including patients and caregivers) in research methods (tailored to the needs of the members and their roles in the study), distributive leadership, and working effectively with patients and caregivers.

***Patients and Caregivers***

Patients and caregivers on the CABs and Patient & Caregiver Ad Hoc Group will receive training, as needed, to ensure they are equipped to undertake their roles on these committees. Training will be tailored to the role of each committee. For example, those in the Patient & Caregiver Ad Hoc group will be responsible for providing input into the selection of outcome measures and will review study materials. Therefore, individuals on this group may require training focused on research and research methods. Patients and caregivers who are members of the CABs on the other hand, will not need the same level of training regarding research methods, but may need training related to the organization and functioning of the health care system.

**Measures & Data Collection**

A summary of the variables and measures being collected as part of the study, and the timing of these measures, can be found in Appendix M.

***A) Effectiveness***: The primary measure of effectiveness is mental functioning, measured by the mental component summary (MCS) score of the Veterans RAND 12 Item Health Survey (VR-12). CESD-10 (23) will be a secondary outcome to determine the presence and severity of depressive symptoms, the GAD-7 (24) will be used to measure anxiety. The Personal Resource Questionnaire (PRQ) will measure perceived social support, and the Patient-Provider Communication, and adapted Client-Centred Care Questionnaire (CCCQ) and Integrated Care Patient-Reported Experience Measure (IC PREMs) will be used to measure patient experience.

The Patient and Caregiver Advisory Committee will assist with the development of an additional patient-relevant and caregiver-relevant outcome measure. The co-development of this measure helps to ensure that outcomes that are relevant and meaningful to patients and caregivers are included. The Patient and Caregiver Advisory Committee co-developed the Unmet Care Needs questionnaire.

Caregivers who participate in the trial will be assessed for caregiver strain (Modified Caregiver Strain Index), depressive symptoms (CESD-10), anxiety (GAD-7) health-related quality of life (VR-12), use of health and social services (HSSUI), and caregiver-relevant measures (Unmet Care Needs, Patient-Provider Communication, and adapted CCCQ and IC PREMs).

*Use & Cost of Health and Community Support Services*: With limited literature on care transition interventions addressing mental health and/or multimorbidity (15), this study will aim to contribute to the broader care transitions literature; therefore, we will utilize commonly reported measures (e.g., mortality, emergency department visits, and hospital readmissions). We will link study participants to ICES administrative databases and follow them for 1 year. Challenges have occurred with the timely linking of trial data with ICES data; therefore, we will also collect service use data from patients and caregivers using our Health and Social Services Utilization Inventory (HSSUI). The HSSUI is a self-report tool that assesses participants’ use of health and community support services over designated periods of time. The HSSUI builds on the work of Browne and colleagues (25, 26). The tool has been assessed for reliability and validity and is one of the few empirically validated measures of health service utilization.

Using the utilization information from the HSSUI along with an Ontario costing manual that has been developed in the Aging, Community and Health Research Unit (ACHRU) in the School of Nursing at McMaster, we will conduct a cost analysis where we will compare the change in the costs of use of health and community support services (including costs associated with the intervention) between participants in the intervention and control groups from baseline to 6 and 12-months.

The cost analysis will also enable us to understand the utilization of services over time within both the intervention and control groups. Thus we can determine the specific services that are most and least affected by the intervention. Taken together, these data will enable us to make informed policy recommendations regarding the implementation of the intervention in Ontario from a cost perspective.

Research assistants will conduct structured interviews with the participants at three time points: baseline (T1), 6 months (T2), and 12 months (T3). These assessments will be completed by phone or in person, depending on participants’ preferences. Each assessment will take approximately one to one and a half hours to complete. The RA will be blinded to group allocation. (Refer to Appendix I for T1 Assessment and Appendix N for T2/T3 Assessment).

Caregivers who participate in the trial will be invited to complete a self-administered questionnaire (See Appendix O) at baseline (T1), 6 months (T2), and 12 months (T3). Caregivers will be given the option to complete the questionnaire in hard copy or by phone. A printed copy of the questionnaire and the Caregiver Information Letter (Appendix AH) will be left by the RA in the patient’s home at the end of each patient interview. The caregiver will be asked to complete the questionnaire and return it to the research coordinator at McMaster University, in a pre-addressed, stamped envelope. Alternatively, the caregiver may choose to complete the questionnaire by phone with a trained interviewer, at a time that is convenient for them. The caregiver’s verbal consent will be obtained prior to completing the questionnaire by phone. The interviewer will mail a copy of the Caregiver’s Letter of Information, to the caregiver, following the interview. The interviewer will maintain a log of verbal consents (date/time) and the caregiver’s study ID.

***B) Implementation of the Intervention***:

We will use both quantitative (implementation outcomes) and qualitative (process of implementation) measures to examine the implementation of the intervention (see Appendix M – Measures and Variables).

*Implementation Outcomes*:

The following implementation outcomes, selected from work by Peters and colleagues (27) will be examined: acceptability, adoption, feasibility, fidelity, and implementation cost (refer to Appendix M for definitions).

*Process of Implementation - Factors Influencing Implementation of the Intervention:*

The Consolidated Framework for Implementation Research (CFIR; 28) will be used to guide data collection and data analysis. As part of the implementation evaluation, the patient/caregiver engagement strategy will be evaluated to examine CAB and Patient/Caregiver Ad Hoc group members’ perceptions of the quality and impact of their involvement, and contextual factors that shape implementation of the patient/caregiver engagement strategy.

*Focus groups and/or interviews*:

i) CABs and Patient/Caregiver Research Partners: Focus groups and online surveys (see Appendix W: Research Partner Questionnaire) will be conducted with the CABs at two time points (mid-way through intervention implementation and after all participants have been recruited at each site). This will allow the researchers to explore the experience of the CABs in implementing the intervention early on in the process and to reflect on the whole experience when recruitment is completed. Focus groups with the CABs will either be held as part the CAB meetings or at a separate time. (This will be determined in consultation with the CAB at each site.) Interviews with Patient and Caregiver Research partners will be conducted at two time points (mid-way through intervention implementation and after all participants have been recruited), to discuss the approaches used to plan and conduct the CAST study; how the program has been implemented and adapted in the three communities; and how patients, caregivers and other community partners have been engaged as partners in the research team (see Appendix AJ: Patient/Caregiver Research Partners – Interview 1 Guide).

*ii) CTCs*: Interviews with the CTCs will occur mid-way through intervention implementation, and after all participants have been recruited (see Appendix P and Appendix Q for interview scripts). This will allow exploration of the intervention at the early, mid and end stages of the trial. Interviews will be conducted by phone by the RC at a time that is convenient for the CTCs.

*iii) Key informants involved in implementing the intervention in collaboration with the CTC (e.g., hospital and community representatives)*: Focus groups will be conducted with the participating hospital and key community organization(s) in each site at 3, 6, and 12 months following the start of the intervention. Focus groups will be scheduled at a time that is convenient for the key informants. Refer to Appendix R for key informant consent form (to be used with CABs, CTCs, and other key informants.

*iv) Patients and caregivers receiving the intervention*: At T2, semi-structured telephone interviews will also be conducted with approximately 7 participants or caregivers per site who were assigned to the intervention group. (See Appendix S for interview guide). These interviews will assess the participants’ and caregivers’ experience of participating in the intervention.

*Meeting minutes*:

Minutes and notes from the CAB, Steering Committee, and research team meetings will also be used to inform understanding of the implementation of the intervention, including some of the implementation outcomes and barriers and facilitators to implementation.

**DATA ANALYSIS**

***A) Effectiveness*:**

*Sample Size Justification and Subgroup Analyses*

The sample size is based on the pilot study for the Mental Health Component Summary (MCS) Score. This assumes power of 80%, alpha of 0.05, mean MCS score difference of 6.5, standard deviation of 15.0, and 20% attrition (based on the pilot).

The ICES data analyses will explore some of the socio-demographic factors (e.g., age, gender, income, rurality) associated with care transitions and service use. Subgroup analyses will be conducted to determine patient groups that benefit most from the intervention. Based on the post-discharge depression and care transition literature, this includes men (3), those living in rural areas (3) or lower income neighbourhoods (2, 3, 29-31), and those with specific chronic conditions (31). Subgroup analyses will explore the extent to which the intervention is effective for various high-risk groups. For example, we will evaluate whether the intervention can successfully address risk factors for a high number of care transitions or high level of burden (measured by service use).

*Effectiveness Outcomes*

The mean difference and its 95% confidence interval will be used to estimate the mean effect of the intervention (compared to usual care) on continuous outcome measures (e.g., re-admission days, CESD-10 and MCS scores). Risk ratios and their 95% confidence intervals will be calculated to determine the effect of the intervention (compared to usual care) on dichotomous outcome measures (mortality, hospital readmissions, and emergency department visits). Since cost data are typically highly skewed, we anticipate calculating median cost differences to compare intervention and usual care costs. Mean effects for continuous and dichotomous outcomes will be captured at key data collection points.

***B) Implementation of the Intervention***:

*Implementation Outcomes*

The implementation outcomes will be analyzed using descriptive statistics (e.g., frequencies, proportions, means, and standard deviations).

*Process of Implementation of the Intervention*

Focus group and interview data, as well as meeting minutes/notes, will be analyzed using conventional content analysis (32). Data will be coded independently by two investigators and a coding list developed. Line-by-line coding will identify key concepts, which will be combined into categories. The investigators will discuss the codes and categories, using a consensus process in identifying overall themes.

**REFERENCES**

1. Bruce ML, McAvay GJ, Raue PJ, Brown EL, Meyers BS, Keohane DJ, et al. Major depression in elderly home health care patients. Am J Psychiatry. 2002 Aug;159(8):1367-74.

2. Neiterman E, Wodchis WP, Bourgeault IL. Experiences of older adults in transition from hospital to community. Can J Aging. 2015 Mar;34(1):90-9.

3. Lin E, Diaz-Granados N, Stewart DE, Bierman AS. Postdischarge care for depression in Ontario. Can J Psychiatry. 2011 Aug;56(8):481-9.

4. Informing the Future: Mental Health Indicators for Canada. Ottawa, ON2015.

5. Smith DJ, Court H, McLean G, Martin D, Langan Martin J, Guthrie B, et al. Depression and multimorbidity: a cross-sectional study of 1,751,841 patients in primary care. J Clin Psychiatry. 2014 Nov;75(11):1202-8; quiz 8.

6. Beverly EA, Wray LA, Chiu CJ, Weinger K. Perceived challenges and priorities in co-morbidity management of older patients with Type 2 diabetes. Diabet Med. 2011 Jul;28(7):781-4.

7. Calderon-Larranaga A, Abad-Diez JM, Gimeno-Feliu LA, Marta-Moreno J, Gonzalez-Rubio F, Clerencia-Sierra M, et al. Global health care use by patients with type-2 diabetes: Does the type of comorbidity matter? Eur J Intern Med. 2015 Apr;26(3):203-10.

8. Jencks SF, Williams MV, Coleman EA. Rehospitalizations among patients in the Medicare fee-for-service program. N Engl J Med. 2009 Apr 2;360(14):1418-28.

9. Forster AJ, Murff HJ, Peterson JF, Gandhi TK, Bates DW. The incidence and severity of adverse events affecting patients after discharge from the hospital. Ann Intern Med. 2003 Feb 4;138(3):161-7.

10. Quality in Primary Care: Setting a Foundation for Monitoring and Reporting in Ontario. Toronto2015.

11. Boyd CM, Boult C, Shadmi E, Leff B, Brager R, Dunbar L, et al. Guided care for multimorbid older adults. Gerontologist. 2007 Oct;47(5):697-704.

12. Bryant-Lukosius D, Carter N, Reid K, Donald F, Martin-Misener R, Kilpatrick K, et al. The clinical effectiveness and cost-effectiveness of clinical nurse specialist-led hospital to home transitional care: a systematic review. J Eval Clin Pract. 2015 Oct;21(5):763-81.

13. Rennke S, Ranji SR. Transitional care strategies from hospital to home: a review for the neurohospitalist. Neurohospitalist. 2015 Jan;5(1):35-42.

14. Rennke S, Nguyen OK, Shoeb MH, Magan Y, Wachter RM, Ranji SR. Hospital-initiated transitional care interventions as a patient safety strategy: a systematic review. Ann Intern Med. 2013 Mar 5;158(5 Pt 2):433-40.

15. Kansagara D, Chiovaro JC, Kagen D, Jencks S, Rhyne K, O'Neil M, et al. 2015 Jan Recommendations for Improving Transitional Care in the Veterans Health Administration.

16. DuGoff EH, Dy S, Giovannetti ER, Leff B, Boyd CM. Setting standards at the forefront of delivery system reform: aligning care coordination quality measures for multiple chronic conditions. J Healthc Qual. 2013 Sep-Oct;35(5):58-69.

17. Hesselink G, Schoonhoven L, Barach P, Spijker A, Gademan P, Kalkman C, et al. Improving patient handovers from hospital to primary care: a systematic review. Ann Intern Med. 2012 Sep 18;157(6):417-28.

18. Allen J, Hutchinson AM, Brown R, Livingston PM. Quality care outcomes following transitional care interventions for older people from hospital to home: a systematic review. BMC Health Serv Res. 2014 Aug 15;14:346.

19. Markle-Reid M, McAiney C, Forbes D, Thabane L, Gibson M, Browne G, et al. An interprofessional nurse-led mental health promotion intervention for older home care clients with depressive symptoms. BMC Geriatr. 2014 May 10;14:62.

20. Markle-Reid MF, McAiney C, Forbes D, Thabane L, Gibson M, Hoch JS, et al. Reducing depression in older home care clients: design of a prospective study of a nurse-led interprofessional mental health promotion intervention. BMC Geriatr. 2011 Aug 25;11:50.

21. Zwarenstein M, Treweek S, Gagnier JJ, Altman DG, Tunis S, Haynes B, et al. Improving the reporting of pragmatic trials: an extension of the CONSORT statement. BMJ. 2008 Nov 11;337:a2390.

22. Curran GM, Bauer M, Mittman B, Pyne JM, Stetler C. Effectiveness-implementation hybrid designs: combining elements of clinical effectiveness and implementation research to enhance public health impact. Med Care. 2012 Mar;50(3):217-26.

23. Radloff LS: The CES-D scale: a self-report depression scale for research in the general population. Appl Psych Meas 1977, 1:385-401.

24. Spitzer RL, Kroenke K, Williams JBW, Lowe B: A brief measure for assessing generalized anxiety disorder: the GAD-7. Arch Intern Med 2006, 166:1092-1097.

25. Browne G, Roberts J, Gafni A, Byrne C, Weir R, Majumdar B, Watt S: Economic evaluations of community-based care: lessons from twelve studies in Ontario. J Eval Clin Pract 1999, 5:367-385.

26. Browne G, Roberts J, Byrne C, Gafni A, Weir R, Majumdar B: The costs and effects of addressing the needs of vulnerable populations: results of 10 years of research. Can J Nurs Res 2001, 33:65-76.

27. Peters DH, Adam T, Alonge O, Agyepong IA, Tran N. Implementation research: what it is and how to do it. BMJ. 2013 Nov 20;347:f6753.

28. Damschroder LJ, Aron DC, Keith RE, Kirsh SR, Alexander JA, Lowery JC. Fostering implementation of health services research findings into practice: a consolidated framework for advancing implementation science. Implement Sci. 2009 Aug 07;4:50.

29. Arbaje AI, Wolff JL, Yu Q, Powe NR, Anderson GF, Boult C. Postdischarge environmental and socioeconomic factors and the likelihood of early hospital readmission among community-dwelling Medicare beneficiaries. Gerontologist. 2008 Aug;48(4):495-504.

30. Piraino E, Heckman G, Glenny C, Stolee P. Transitional care programs: who is left behind? A systematic review. Int J Integr Care. 2012 Jul-Sep;12:e132.

31. Kadam UT, Uttley J, Jones PW, Iqbal Z. Chronic disease multimorbidity transitions across healthcare interfaces and associated costs: a clinical-linkage database study. BMJ Open. 2013;3(7).

32. Hsieh HF, Shannon SE. Three approaches to qualitative content analysis. Qual Health Res. 2005 Nov;15(9):1277-88.

**LIST OF ABBREVIATIONS**

| **ACHRU** | Aging, Community and Health Research Unit |
| --- | --- |
| **CAB** | Community Advisory Board |
| **CAST**  **CESD-10** | Community Assets Supporting Transitions  Centre for Epidemiological Studies on Depression (10 question version) |
| **CTC** | Care Transition Coordinator |
| **HGH** | Hamilton General Hospital |
| **HSN** | Health Sciences North |
| **HSSUI** | Health and Social Services Utilization Inventory |
| **ICES** | Institute for Clinical Evaluative Sciences |
| **JBH** | Joseph Brant Hospital |
| **MCC** | Multiple Chronic Conditions |
| **MCS** | Mental Health Component Summary Score (from SF-12) |
| **PHQ-2** | Patient Health Questionnaire, Version 2 |
| **PROMIS** | Patient Records and Outcome Management Information System |
| **RA** | Research Assistant |
| **RC** | Research Coordinator |
| **RCT** | Randomized Controlled Trial |

**REVISION HISTORY**

*Version Number: 1.0*

Version Date: November 29, 2016

*Version Number: 2.0*

Version Date: May 9, 2017

Summary of Revisions Made:

- Changes to recruitment procedure
- Option provided for in-home consent and baseline visit
- Protocol for contacting patients and identifying patients “lost to follow-up” expanded
- Audio-recording of telephone interviews added
- Intervention dose clarified (maximum/minimum home visits and phone calls)
- Administrative revisions and minor changes made for clarity
- Addition of Appendix T: Hospital Recruiter Role
- Addition of Appendix U: Graphical Depiction of Trial Components
- Addition of Appendix V: Study Information Letter
- Addition of Appendix W: Key Informant Questionnaire
- Addition of Appendix X: CAB Interview 1 Guide
- Revisions to the following Appendices:
  - Appendix A: Governance Structures
  - Appendix B: Qualifying Comorbid Conditions
  - Appendix C: Study Timeline
  - Appendix D: Participant Flow Diagram
  - Appendix E: Screening Script
  - Appendix F: Eligibility Questionnaire
  - Appendix G: Participant Contact Information Form
  - Appendix H: Participant Consent Form
  - Appendix I: Participant Interview, T1
  - Appendix J: Research Assistant Telephone Scripts
  - Appendix N: Participant Interview, T2/T3
  - Appendix O: Caregiver Self-Administered Questionnaire
  - Appendix R: Key Informant Consent Form

*Version Number: 3.0*

Version Date: June 22, 2017

Summary of Revisions Made:

- ClinicalTrials.gov ID added
- GAD-7 added to caregiver assessment
- Patient experience measures added to patient and caregiver assessments: Patient-Provider Communication, and Adapted CCCQ and IC PREMs
- Patient and caregiver-co-developed measure added to patient and caregiver assessments: Unmet Care Needs
- Assessment data are to be inputted electronically by the RA into LimeSurvey software
- Names and contact information of newly-enrolled study participants are to be shared by the RC with the CTC using MacDrop software
- Addition of Appendix Y: Appointment Reminder Card
- Revisions to the following Appendices:
  - Appendix F: Eligibility Questionnaire
  - Appendix H: Participant Consent Form
  - Appendix I: Participant Interview, T1
  - Appendix N: Participant Interview, T2/T3
  - Appendix O: Caregiver Self-Administered Questionnaire

*Version 4.0*

Version Date: November 2, 2017

Summary of Revisions Made:

- Rebecca Ganann added to list of co-investigators
- Timing of CAB, Steering Committee, and CTC data collection points revised to “mid-way through intervention implementation, post-intervention implementation, and 6 months following the end of intervention implementation at each site.”
- Addition of Appendix Z: Steering Committee Interview 1 Guide
- Revisions to the following Appendices:
  - Appendix I: Participant Interview, T1
  - Appendix L: Nurse Telephone Script
  - Appendix M: Variables and Measures
  - Appendix N: Participant Interview, T2/T3
  - Appendix P: CTC Interview 1 Guide
  - Appendix X: CAB Interview 1 Guide

*Version 5.0*

Version Date: February 6, 2018

Summary of Revisions Made:

- Tracey Chambers added as Research Coordinator
- Contact information for McMaster University Principal Investigator, Dr. Carrie McAiney, updated
- Revisions to the following Appendices:
  - Appendix F: Eligibility Questionnaire
  - Appendix G: Participant Contact Information Form
  - Appendix H: Participant Consent Form
  - Appendix O: Caregiver Self-Administered Questionnaire
  - Appendix R: Key Informant Letter of Information and Consent Form
  - Appendix T: Hospital Recruiter Role
  - Appendix V: Research Study Information Sheet
- Addition of Appendix AA: Physician Letter
- Addition of Appendix E: Recruiter Screening Script – French
- Addition of Appendix F: Eligibility Questionnaire – French
- Addition of Appendix G: Participant Contact Information Form – French
- Addition of Appendix H: Participant Consent Form – French (Sudbury site)
- Addition of Appendix I: Participant Interview, T1 – French
- Addition of Appendix J: Research Assistant Telephone Scripts – French
- Addition of Appendix L: Nurse Telephone Script – French
- Addition of Appendix O: Caregiver Self-Administered Questionnaire – French
- Addition of Appendix V: Study Information Sheet – French
- Addition of Appendix Y: Appointment Card Reminder - French

*Version 6.0*

Version Date: May 17, 2018

Summary of Revisions Made:

- List of Co-Investigators and Trainees updated
- Role of caregivers as trial participants defined and clarified
- Process of obtaining a participant’s informed consent, and the role of the substitute decision-maker, (i.e. caregiver) described in greater detail
- Process of collecting data from caregivers, via self-administered questionnaire, described in greater detail.
- Personal resource questionnaire (PRQ) (previously omitted in protocol text) identified in protocol text
- Revisions to the following Appendices:
  - Appendix H: Participant Consent Form
  - Appendix J: Research Assistant Telephone Scripts
  - Appendix O: Caregiver Self-Administered Questionnaire
- Addition of Appendix AH: Caregiver Letter of Information

Version 7.0

Version Date: August 27, 2018

Summary of Revisions Made:

- Institution/Location of Co-Principal Investigator, Carrie McAiney, updated to University of Waterloo.
- Designation for Co-Principal Investigator, Dr. Maureen Markle-Reid, changed from Associate Professor to Professor
- Research Coordinator, Tracey Chambers, removed. New Research Coordinator to be hired.

Revisions to Protocol:

- Removed Steering Committee
- Removed participant written consent at 6-month and 12-month assessments.
- Changed number of focus groups with community advisory board members from 3 to 2.
- Changed number of interviews with care transition coordinators from 3 to 2.
- Added description of interviews with patient/caregiver research partners.
- Revisions to the following Appendixes:
  - Appendix H: Participant Letter of Information and Consent Form (Hamilton/Burlington sites)
  - Appendix I: Participant Data Collection Form – Timepoint 1
  - Appendix N: Participant Data Collection Form – Timepoint 2 and Timepoint 3
  - Appendix O: Caregiver Self-Administered Questionnaire
  - Appendix Q: Care Transition Coordinator Interview 2 Guide
  - Appendix R: Key Informant Letter of Information/Consent Form
  - Appendix AA: Physician Letter
  - Appendix AC: Participant Consent to Share Information – CTC & Circle of Care (Hamilton/Burlington sites)
  - Appendix AD: Participant Consent to Share Information – CTC Referrals (Hamilton/Burlington sites)
  - Appendix AF: Participant & Caregiver Consent Form – Post-Intervention Interview
  - Appendix AG: Research Partner Questionnaire Email Invitation
  - Appendix AH: Caregiver Letter of Information
- Addition of:
  - Appendix AI: Community Advisory Board Guide 2
  - Appendix AJ: Patient/Caregiver Research Partners – Interview 1 Guide
